# Role of Metabolic Reprogramming in Epithelial–Mesenchymal Transition (EMT)

**DOI:** 10.3390/ijms20082042

**Published:** 2019-04-25

**Authors:** Hyunkoo Kang, Hyunwoo Kim, Sungmin Lee, HyeSook Youn, BuHyun Youn

**Affiliations:** 1 Department of Integrated Biological Science, Pusan National University, Busan 46241, Korea; kanghk94@gmail.com (H.K.); harlemkim@gmail.com (H.K.); smlee1048@gmail.com (S.L.); 2 Department of Integrative Bioscience and Biotechnology, Sejong University, Seoul 05006, Korea; hsyoun@sejong.ac.kr; 3 Department of Biological Sciences, Pusan National University, Busan 46241, Korea

**Keywords:** metabolic reprogramming, EMT, metastasis, cancer progression

## Abstract

Activation of epithelial–mesenchymal transition (EMT) is thought to be an essential step for cancer metastasis. Tumor cells undergo EMT in response to a diverse range of extra- and intracellular stimulants. Recently, it was reported that metabolic shifts control EMT progression and induce tumor aggressiveness. In this review, we summarize the involvement of altered glucose, lipid, and amino acid metabolic enzyme expression and the underlying molecular mechanisms in EMT induction in tumor cells. Moreover, we propose that metabolic regulation through gene-specific or pharmacological inhibition may suppress EMT and this treatment strategy may be applied to prevent tumor progression and improve anti-tumor therapeutic efficacy. This review presents evidence for the importance of metabolic changes in tumor progression and emphasizes the need for further studies to better understand tumor metabolism.

## 1. Introduction

It is widely accepted that tumorigenesis is initiated by gain-of-function of oncogenic genes and loss-of-function of tumor suppressor genes in normal cells, which as a result, show loss of normal function and uncontrolled proliferation [1]. Especially, the mutation in tumor suppressive genes are more significantly involved in tumorigenesis and Tumor protein p53 (TP53), Breast cancer type 1/2 susceptibility protein (BRCA1/2), and Phosphatase and tensin homolog (PTEN) which protect DNA from mutation accumulation have been suggested as frequently mutated tumor suppressor genes in tumor cells [2]. During this process, therefore, cancer cells often show an altered metabolic status and some of these metabolic disturbances following altered metabolic gene regulation have been shown to be common among different types of tumor cells. The investigation of metabolic reprogramming, a hardwired metabolic shift in tumor cells, is thought to be useful for understanding the metabolic state of tumor cells [3]. Most tumor cells showed highly activated glycolysis, through increased expression of glycolytic enzymes or the expression of enzyme isoforms that are not expressed in normal differentiated cells [4]. This phenomenon often leads to a high respiratory capacity and the activation of proliferative signaling pathways in tumor cells. Moreover, tumor cells utilize glycolysis for energy production, despite their surrounding aerobic environment. This is known as the Warburg effect [5]. The significance of the Warburg effect has been investigated in many studies and it has been widely targeted for the development of improved anti-tumor therapies. In addition, recent studies have also reported other metabolic alterations in tumor cells, such as changes in glutamine utilization for the enhancement of glycolytic pathways and highly activated lipid synthetic pathways [6,7]. Although the roles of altered metabolic pathways in tumors have been widely investigated, the specific mechanisms of tumor metabolism are not yet fully understood.

Many studies have investigated metabolic reprogramming in cancer cells and have suggested some driving forces that account for metabolic reprogramming [8,9]. A close relationship between oncogenic mutations and the occurrence of metabolic reprogramming has been reported in many studies [10]. Recent studies have broadened this concept by showing that mutations and epigenetic changes that occur during tumor development processes can also lead to metabolic reprogramming [11,12]. Conversely, it has also been suggested that altered metabolism increases the vulnerability of cancer cells to mutagenesis and epigenetic alteration [13,14]. These studies suggest that somatic mutations, epigenetic alterations, and metabolic reprogramming form a feed-forward cycle that leads to tumor malignancy. Therefore, a better understanding of these events in tumor cells is needed to develop strategies to halt this vicious cycle and suppress cancer progression.

Epithelial–mesenchymal transition (EMT) is a type of cellular differentiation that can be easily observed during developmental processes. Through the regulation of EMT, systems, organs, and tissues are formed, and their function, growth, and regeneration are maintained [15]. However, dysregulated EMT can cause diseases, including maldevelopment, fibrosis, and neoplastic progression [16]. In tumor cells, EMT is thought to be an important step preceding invasion and metastasis, and a significant role of EMT in tumor progression has been reported in many studies [17]. EMT induction is mediated by oncogenic signal transduction, involving the PI3K-AKT-mTOR, EGFR-RAS-MAPKs, and JAK2-STAT3 pathways [18,19]. In addition, some transcription factors, including Zinc finger protein SNAI1 (SNAIL), Zinc finger protein GLI1/2 (GLI1/2), Twist-related protein (TWIST), and Zinc finger E-box-binding homeobox 1 (ZEB1/2) are also reportedly involved in EMT induction [17,20]. Although some preclinical studies suggested key factors that account for EMT and invasiveness induction, it could not completely control the tumor progression and metastasis [21,22]. Although novel techniques have recently been attempted to regulate EMT in tumor cells, the fundamental control of EMT in tumor cells has not been yet been achieved.

In this review, we focus on metabolic alterations in tumor cells and their role in EMT induction. We discuss alterations in enzymes involved in glucose, lipid, and amino acid metabolism and their underlying molecular mechanisms in EMT induction. In addition, we discuss the clinical potential of metabolic enzyme-specific inhibitors for the suppression of tumor metastasis.

## 2. Glucose Metabolism in EMT

Metabolic rewiring towards an enhanced glycolytic phenotype plays a critical role in tumor aggressiveness. This mainly involves an increase in glucose uptake and glycolysis flux, mitochondrial dysfunction, and a more acidic tumor microenvironment. The interaction between the tumor microenvironment and EMT induction has emerged in recent studies [23,24]. Through these metabolic changes, cancer cells may adopt cancer stem cell (CSC)-like properties and express EMT-inducing transcription factors. In this section, we focus on the molecular mechanisms, whereby reprogrammed metabolism promotes tumor metastasis.

An increase in glucose transporter expression is one of the major causes of enhanced glucose metabolism in malignant tumors. Among these transporters, GLUT1 and GLUT3, which are induced by hypoxia-inducible factor 1α (HIF-1α), have been shown to increase glycolysis and cancer progression [25]. Overexpression of GLUT1 increases matrix metalloproteinase 2 (MMP-2) expression *in vitro* and *in vivo*, which is essential for EMT and cellular invasiveness [26,27]. In pancreatic cancer cells, GLUT1 directly regulates MMP-2 expression levels and promoter activity [27]. Although GLUT1 is a major glucose transporter in many cancer cells, GLUT3 also contributes to glucose uptake and cancer progression [28,29]. In non-small cell lung cancer, GLUT3 is highly expressed in mesenchymal cells but not in epithelial cells. Moreover, ZEB1, an EMT marker, transcriptionally activates the *GLUT3* gene and GLUT3 expression correlates with poor patient survival [28]. Brain tumor-initiating cells, which act as CSCs in brain cancer, preferentially uptake glucose using GLUT3, while most other cells use GLUT1 [29]. This feature makes CSCs more aggressive and persistent in glioblastoma. In summary, glucose transporters, especially GLUT1 and GLUT3, promote tumor progression by increasing glucose influx and activating downstream molecular pathways.

Hexokinase catalyzes the phosphorylation of glucose to glucose-6-phosphate, which is the first step of glycolysis. In cancer, hexokinase 2 (HK2) is often upregulated as a result of augmented glucose metabolism [30]. Lung cancer initiation and progression are significantly inhibited in *Hk2*-knockout mice, whereas the upregulation of HK2 can induce EMT due to metabolic reprogramming. The expression of HK2 is significantly increased in brain metastatic derivatives of breast cancer cells [31]. HK2 is a well-known hypoxia-inducible gene that can induce EMT. Increased expression of HK2 has been shown to promote EMT both *in vitro* and *in vivo* through an enhanced glycolytic phenotype under hypoxic conditions in tongue squamous cell carcinoma [32]. A hypoxic environment increases HK2 expression and the migration and invasion ability of tongue squamous cell carcinoma cells. In the same context, knockdown of HK2 results in decreased cell migration and invasion, whether in a hypoxic or normoxic environment. In addition, either hypoxia or the upregulation of HK2 increases the expression of the stem cell markers, SNAIL and SLUG. microRNA-155 (miR-155) and miR-143 have also been shown to regulate *HK2* [33]. miR-143 inhibits cell migration and metastasis both *in vitro* and *in vivo* by targeting *HK2* and miR-155 represses miR-143 and activates Signal transducer and activator of transcription 3 (STAT3), an activator of HK2. Phosphofructokinase (PFK), the rate-limiting enzyme of glycolysis, is also induced by HIF-1α and increases glucose uptake. Increased expression of PFK increases glycolytic flux and EMT by maintaining this glycolytic phenotype in cancer cells *in vitro* [34]. However, a high level of PFK expression does not always have a positive influence on cancer cell survival and proliferation. Under nutrient restriction, cancer cells alter glycolytic flux to pentose phosphate pathway to overcome oxidative stress by suppressing PFKP [35]. Long-term hypoxic conditions may also inhibit PFK by O-GlcNAcylation [36]. Suppressed PFK1 activity reduces the glycolytic rate and ATP levels and induces tumor metastasis both *in vitro* and *in vivo* in an adverse microenvironment. Pyruvate kinase is the enzyme that catalyzes the final step of glycolysis. Pyruvate kinase M2 (PKM2), which results from a splice variant of the pyruvate kinase gene, regulates glucose metabolism in cancer cells and induces tumorigenesis [37]. PKM2 enhances cell survival and invasion by increasing glucose uptake and lactate production in pancreatic ductal adenocarcinoma *in vitro* and *in vivo* [38]. PKM2 can induce EMT through metabolic mechanisms, but also through nonmetabolic mechanisms via nuclear translocation [39]. Nuclear PKM2 binds to TGFB induced factor homeobox 2 (TGIF2), which is a transcription factor of Cadherin-1 (*CDH1)*, and represses *CDH1* transcription and stimulates the mesenchymal marker, vimentin, during EMT both in *in vitro* and *in vivo* colorectal cancer models. Furthermore, in hepatocellular carcinoma (HCC) cells, ERK-mediated nuclear translocation of PKM activates β-catenin *in vitro*, which induces EMT through the transcription factor, T-cell factor/lymphoid enhancer-binding factor (TCF-LEF) [40]. Highly-metastatic HCC cells express high levels of PKM2 *in vitro* and induce infiltration of myeloid-derived suppressor cells to metastatic nodules *in vivo* [41]. Therefore, these upregulated glycolytic enzymes induce EMT as well as glycolysis.

AMP-activated protein kinase (AMPK) is a serine/threonine protein kinase that is activated under conditions of low energy, such as glucose starvation and hypoxia [42]. Liver kinase B1 (LKB1), which phosphorylates and activates AMPK, is a well-known tumor suppressor [43]. LKB1/AMPK signaling downregulates SNAIL and ZEB1, which are the EMT marker proteins, and inhibits the invasion and migration of tumor cells, by regulating signaling pathways, such as those involving NF-κB, AKT, FOXO3, TGF-β, and mTOR. In clinical prostate cancer samples, phospho-AMPK expression is highly elevated and the activation of AMPK has been shown to increase cellular motility and invasion in human prostate cancer cell lines [44]. Moreover, some recent studies have indicated that AMPK can induce invasion and metastasis in cancer cells. ARK5 is one of the AMPK catalytic subunits that are directly activated by AKT. It protects cancer cells from glucose deprivation by inhibiting caspase 8 activation both in *in vitro* and *in vivo* models [45]. AMPK-related kinase 5 (ARK5) also increases the invasive activity of pancreatic cancer cells, and overexpression of ARK5 significantly enhances tumorigenicity. Furthermore, in *Pten*-deficient thyroid cancer, PI3K/Akt-mediated AMPK inhibition decreases oxidative phosphorylation and induces thyroid hyperplasia both in *in vitro* and *in vivo* models [46]. In summary, although AMPK is known to negatively regulate cancer cell proliferation and progression, it can have a dual function, whereby it also induces tumor invasion and metastasis, depending on the context.

The effect of altered mitochondrial function on EMT induction has been the focus of numerous studies. In the breast cancer cell line, an increase in the invasiveness of cell lines is associated with impaired mitochondrial function and a decreased sensitivity to mitochondrial inhibitors [47]. Moreover, both *in vitro* and *in vivo* invasive breast cancer cells show a loss of transmembrane protein 126A (TMEM126A), mitochondrial retrograde signaling alterations, EMT induction, and altered extracellular matrix composition [48]. An *in vitro* study using TGF-β1 to inhibit mitochondrial function also supports the involvement of mitochondrial dysfunction in EMT induction [49]. Furthermore, mitochondrial DNA (mtDNA)-depleted HCC cell line have significantly increased motility and invasive capacity mediated by the TGF-β/SMAD/SNAIL signaling pathway [50]. Mitochondrial dysfunction also induces the phosphorylation of c-Jun/AP-1, which is known as an activator of TGF-β. Disruption of the electron transport chain results in a loss of mitochondrial membrane potential and increased cytosolic Ca^2+^ levels [51], leading to the induction of calcineurin Aα (CnAα)-mediated mitochondrial retrograde signaling both in *in vitro* and *in vivo* models [52]. Accordingly, silencing CnAα in mtDNA-depleted breast cancer cells reduces the expression of EMT-related proteins, such as N-cadherin, vimentin, and SNAIL. Similarly, in mtDNA-depleted myoblast cells, CnAα knockdown significantly decreases the expression of Glut4 and IGF1R. CnAα is thought to be upstream of the proteins that induce glucose uptake and resistance to apoptosis [53]. Consequently, the activation of CnAα by mtDNA depletion induces both a metabolic shift to glycolysis and invasiveness and anti-apoptotic phenotypes in cancer cells.

Tumor cells secrete acidic byproducts of respiratory processes. Representatively, lactic acid is produced from pyruvate and NADH to replenish NAD^+^ for glycolysis. As a result, tumors are surrounded by an acidic microenvironment, which has been reported to induce EMT in tumor cells. Extracellular lactate induces EMT in lung cancer cells through the activation of SNAIL, which in turn, remodels the extracellular matrix and activates TGF-β1 [54]. The involvement of lactate in EMT has also been demonstrated in a study showing that disintegrin and metalloproteinase (ADAM) 10- and 17-mediated lactate production significantly induced EMT in colorectal cancer cells [55]. EMT induction by acidification may be related to the intracellular capacity for lactate production. The upregulation of mitochondrial topoisomerase I (TOP1MT), observed in invasive cancer cells, increases both *in vitro* and *in vivo* expression of lactate dehydrogenase A (LDHA), which is associated with enhanced glycolysis and EMT in gastric cancer cells [56]. Although the precise roles of acidification and lactate production in the induction of EMT are not yet fully understood, a recent study has provided some insight. In prostate cancer cells, uptake of lactate by MCT1 is enhanced by the cooperation of cancer-associated fibroblasts and the activation of HIF-1α, which promotes cancer progression [57]. These results show that lactate production induced by shifted metabolism in cancer cells may eventually result in enhanced EMT. EMT-inducing stimuli also accelerate acidification of the microenvironment by inducing metabolic changes. Twist, a representative EMT-inducing transcription factor is upregulated in invasive cancer cells and strengthened the glycolytic metabolic pathway by inducing LDHA expression [58]. Taken together, an acidic tumor microenvironment has a significant role in inducing EMT and promoting the invasion of tumor cells.

In summary, malignant tumor cells alter their glucose metabolism to enhance aerobic glycolysis, so that they can maintain their metastatic potential. This metabolic alteration sustains the Warburg effect and induces EMT by enhancing glycolysis and blocking the TCA cycle.

## 3. Lipid Metabolism in EMT

As discussed in Section 1, tumor cells show abnormal lipid metabolism, including both increased lipogenesis and lipolysis. Although the underlying molecular mechanisms are not yet fully understood, previous studies have suggested that the principal enzymes involved in lipid metabolism can alleviate tumor progression. Fatty acids are produced in mammalian cells by using acetyl CoA as the base and repeatedly adding malonyl CoA. Acetyl CoA carboxylase (ACC) is involved in the production of malonyl CoA, which is a key rate-limiting step in fatty acid production [59]. In many studies, ACC has been proposed as a promising target for the inhibition of tumor growth through the regulation of altered metabolism in cancer cells [60,61]. In addition, ACC is activated in the mitochondria by excessive levels of citrate, which is produced by citrate synthase [62]. As citrate synthase expression is upregulated in malignant cancer cells and is essential for growth and proliferation, ACC activity may also be high in cancer cells [63,64]. In the context of EMT induction, the inhibition of fatty acid synthetic enzymes, including fatty acid synthase (FASN), 3-hydroxy-3-methylglutaryl-CoA reductase (HMGCR), and ACC, by miRNA has been reported to suppress EMT in breast cancer cell lines [65]. Conversely, another study has suggested that ACC is inactivated by phosphorylation in invading breast cancer cells, resulting in high acetyl CoA levels, which can then participate in epigenetic gene regulation through the acetylation of SMAD2 in both *in vitro* and *in vivo* models [66]. Although the underlying molecular mechanisms of ACC in EMT is still elusive, a study with kidney proximal tubular cells (HK-2 cell line) suggested that increased lipid content and decreased β-oxidation of fatty acid induced EMT related gene expression, which was alleviated by ACC2-specific silencing [67]. This study provided an insight that lipid contents in tumor cells may mediate between lipogenic metabolic changes and EMT induction. Based on these studies, activated ACC is involved in the high energy utilization of tumor cells and helps the EMT process, while regulating acetylation potential for other molecular events.

After the production of building blocks for fatty acid synthesis by ACC, downstream processes are mediated by a single polypeptide, FASN, which adds malonyl CoA to the acyl group for elongation [68]. A role of FASN in EMT has been widely reported in previous studies of various types of cancer [69,70,71]. In a study investigating molecular mechanisms, FASN was shown to induce EMT through the expression of TGF-β both in *in vitro* and *in vivo* non-small cell lung cancer models [72]. In the same study, EMT induction, in turn, promoted FASN expression to create a positive feedback loop that accelerated cancer progression. FASN has also been reported to sustain the *in vivo* stemness of CSCs by modulating cytoskeleton protein expression [73]. These studies have shown that FASN is an upstream regulator of well-known EMT-inducing factors. Some studies have suggested a relationship between FASN-induced EMT and proliferative signaling proteins, including EGFR and mTOR, which was confirmed by patient-derived tissue analysis [70,74]. It has also been reported that the inhibition of FASN results in suppression of the AKT/mTOR signaling pathway in breast cancer cell line [75]. These studies also indicate that FASN-induced EMT is related to signaling pathways that activate proliferation. The majority of studies have reported that FASN induces EMT in cancer cells; however, some studies have shown that FASN-induced EMT can be inhibited in breast and lung cancer cells by rewiring the metabolic state of the cells [76,77]. As these studies have typically investigated the EMT-inducing role of FASN in tumor cells stimulated by TGF-β1 or hyperglycemia, it can be concluded that FASN has the potential to initialize EMT in most tumor cells, while the downregulation of FASN in transforming tumor cells may accelerate the EMT process.

Lipids are highly charged energy sources that are mainly stored as triacylglycerol (TG) in intracellular lipid droplets. TG is degraded to fatty acids and glycerol upon lipolytic stimuli. Fatty acids are used for energy production by β-oxidation processes in mitochondria, and they supply large quantities of acetyl CoA and ATP [78]. Carnitine palmitoyltransferase 1 (CPT-1), which translocates fatty acids from the cytosol to the mitochondrial interspace for fatty acid oxidation, has been reported to be upregulated in various types of tumor cells and has been suggested as a therapeutic target in previous studies [79,80]. However, the precise role of CPT-1 in EMT induction has not yet been reported. Instead, a study suggesting that prostate cancer cells undergoing EMT show increased TG accumulation indicates a possible negative effect of fatty acid oxidation on EMT induction [81]. Other fatty acid synthesis/oxidation balance regulators, the peroxisome proliferator-activated receptor (PPAR) family members, are activated by fatty acid ligands and act as transcription factors. Their target genes are also involved in lipid synthesis or degradation and the activation of related signaling pathways [82]. In the context of EMT induction, antagonist inhibition of PPARβ/δ induces EMT in melanoma cells, and PPARβ/δ knockout increases the metastasis of melanoma cells *in vivo* [83]. Pharmacological suppression of PPARα/γ has also been reported to induce EMT in normal fibroblasts, in a PPAR activity-dependent manner [84,85]. Induction of PPARγ expression by small molecules has been reported to prevent EMT through the suppression of fibrotic gene expression in a mouse colorectal fibrosis model [86]. In prostate cancer cells, PPARγ deficiency results in the induction of EMT and stemness [87]. These studies suggest that PPAR family members broadly suppress EMT progression and that altered tumor metabolic processes result in abrogated PPAR activity. As an upstream molecular regulator of PPARγ, ERK, a representative proliferative factor, was suggested and inhibition of ERK inhibited PPARγ activation and EMT induction [88]. This relationship between proliferation and PPARγ inhibition was also supported by a *in vivo* study stating cyclin-dependent kinase 5 (CDK5)-mediated EMT induction [89]. However, the precise role of PPARγ in regulation of EMT-related gene expression is not fully understood. Instead, it can be concluded that fatty oxidation negatively regulates EMT induction in cancer cells, which is consistent with FASN-induced EMT induction, as previously discussed.

Another major role of lipids in cellular homeostasis is their function in the plasma membrane. Membranous lipids are essential for cell growth and division, as indicated by the high level of expression of lipid synthetic membrane proteins during proliferation and development [82]. The cellular membrane mostly consists of phosphatidylcholine. Its synthesis is initiated by CTP-phosphocholine cytidylyltransferase (CTT), which is a rate-limiting enzyme in the synthesis process [90]. Upregulation of CTT has been reported to contribute to tumor growth and malignant transformation in breast cancer; however, its precise molecular mechanisms have not yet been investigated [91]. Instead, reports that cells undergoing EMT have increased phosphatidylcholine content and that phosphatidic acid synthesis helps maintain the stemness of cancer cells, have supported the potential role of phospholipids in EMT induction [92,93,94]. Sphingolipid, another membranous lipid with a sphingosine backbone, has been widely reported to be involved in EMT induction. A significant role of sphingosine kinase 1 (SPHK1), which converts sphingosine to sphingosine 1-phosphate (S1P), has been demonstrated in EMT induction in various types of cancer. Treatments that modulate SPHK1 expression, such as miRNA, atorvastatin, and ectopic overexpression, have confirmed that SPHK1 induces EMT in various types of cancer cell lines [95,96,97]. In addition to the biogenesis of membranous lipids, some studies have investigated the underlying molecular events mediated by SPHK1. In hepatic cancer cells, SPHK1 has been reported to induce EMT through TNF receptor associated factor 2 (TRAF2)-mediated autophagic degradation of E-cadherin [98]. Another study reported that SPHK1 activates focal adhesion kinase and induces EMT in colorectal cancer cell lines [99]. Moreover, a study of S1P levels has provided support for the role of sphingolipids in EMT induction. A transcriptomic study of genes related to sphingolipid synthesis, EMT, and the lipidome in cancer cells has suggested a significant correlation between sphingolipid metabolism and EMT induction, especially in lung, bladder, colorectal, and prostate cancer [100]. Other studies have also provided evidence for the role of S1P in the molecular mechanism of EMT induction. S1P treatment has been shown to induce EMT through MMP-7 expression and TGF-β secretion both *in vitro* and *in vivo* models [101]. The molecular mechanisms, whereby S1P induces EMT in cancer cells is not fully understood, but some studies have reported that Rho kinase and SNAIL-MMP signaling pathways are downstream of S1P [102,103]. Taken together, both SPHK1 and S1P are promising targets for the control of EMT in cancer cells and further research on their molecular mechanisms is warranted. Finally, studies of ceramide have further emphasized the role of membranous lipids in EMT induction. Unlike phosphatidylcholine and sphingolipids, ceramides increase the rigidity of the plasma membrane and reduce cell motility [104]. Ceramide synthase, which produces ceramide from sphinganine, is reported to be downregulated during EMT processes and reduced production of C16-ceramides results in EMT inhibition in various cancer cell lines [105]. Taken together, the altered membranous lipid metabolism modulated cell motility, and their metabolic enzymes need to be investigated in further studies.

Several lipids are known to act as signaling molecules through the activation of receptors or scaffold proteins. Tumor cells have been shown to have dysregulated production of lipid signaling molecules, and this induces EMT in some cancer cells. Phosphatidylinositol, a membranous phospholipid, is phosphorylated by phosphatidylinositol kinases and converted into phosphatidylinositol phosphate. Phosphoinositol-4,5-bisphosphate (PIP_2_) is a substrate of PI3K, which catalyzes its conversion to phosphoinositol-3,4,5-triphosphoate (PIP_3_). PIP_3_ mediates tumor progression, including invasive differentiation, through the AKT-mTOR signaling pathway [106]. Along with PI3K signaling pathway activation in tumors, increased production of phosphatidylinositol due to elevated expression of phosphatidylinositol synthase is observed in oral squamous cancer patients [107]. Although this has not been reported in other cancer types, further studies are required to determine how phosphatidylinositol synthase might regulate EMT in cancer cells. Eicosanoids are bioactive lipids derived from arachidonic acids and they have been suggested as promising targets for anti-cancer therapies [108]. Eicosanoids are produced by cyclooxygenase (COX), lipoxygenase (LOX), and cytochrome P450 (CYP450) from arachidonic acid and each lipid derivative activates various cellular signaling pathways [109]. Prostaglandin E_2_ (PGE_2_) is a representative product of COX-2, which can significantly affect EMT in breast cancer cells when its expression is upregulated [110]. An increase in COX-2 expression and a subsequent increase in intracellular PGE_2_ levels have been reported in malignant tumor cells, and many previous studies have indicated that these factors have roles in EMT induction [111,112]. The significant roles of COX-2 and PGE_2_ in EMT induction were confirmed in a study of pharmacological COX-2 inhibitors in breast and ovarian cancer cells [113,114]. In particular, COX-2 was associated with inflammatory signaling pathway activation, and it mediated intercellular communication between macrophages and tumor cells undergoing EMT [112,115]. Roles for members of the LOX family, 12-LOX and 15-LOX, in EMT induction have also been reported, with clinical significance in gastric and prostate cancer patients. These data implicate LOX family members as promising therapeutic targets [116,117]. However, 5-LOX catalyzes the conversion of eicosanoid to lipoxin A_4_, a well-known suppressor of EMT in tumor cells [118,119,120]. These effects are mediated by lipoxin receptors, and studies have suggested that lipoxin A_4_ has a promising role in the suppression of cancer metastasis. CYP450 family members are also critically involved in tumorigenesis and tumor growth, but little is known about the role of CYP450 in tumor EMT [121].

As discussed above, tumor cells show dysregulated lipid metabolism, including high lipogenic and low lipolytic capacity, elevated membranous lipid synthesis, and upregulation of bioactive lipid production, which induces EMT processes. In addition, studies that control EMT induction by regulating lipid metabolism have suggested the need for further studies of tumor lipid metabolism to develop more effective anti-tumor therapies.

## 4. Amino Acid Metabolism in EMT

As we mentioned in Section 2 and Section 3, glucose and lipid metabolism are crucial to not just cancer cells but also normal cells. In addition, amino acid metabolism also has a critical role in maintaining cellular metabolic homeostasis too. Although the underlying molecular mechanisms are not yet fully understood, previous studies have suggested that the principal enzymes involved in amino acid metabolism can alleviate tumor progression. Metabolism of natural amino acids contributes to the migration and invasion of cancer cells. In this section, we summarize several metabolic pathways for amino acids and the mechanisms of EMT regulation by altered amino acid metabolism. Among all amino acids, glutamine has the greatest consumption, and it is now considered the most important substrate in cancer cells. Numerous studies have demonstrated an essential role for glutamine in the biosynthesis of nucleotides and non-essential amino acids and in providing substrates for the TCA cycle to fuel tumor growth [122]. All these processes begin with glutaminolysis. Glutaminolysis has been proposed to be as important as glucose metabolism in tumors, and it is primarily induced by the MYC oncogene [123]. The involvement of glutamine in the regulation of EMT induction has been reported in previous studies, which showed that the inhibition of glutaminolysis, by targeting glutaminase 1 (GLS1), impairs *in vivo* metastasis through the repression of SNAIL [124]. Conversely, the expression of GLS2, the mitochondrial isoform of glutaminase, inversely correlates with tumor stage, tumor size, and prognosis in HCC [125]. It was suggested the molecular mechanism of GLS2 inhibition-mediated tumor suppression was activity-independent stabilization of the EMT-related microRNA, miR-34a both *in vitro* and *in vivo*. These results imply that the involvement of glutamine catabolism in EMT induction may be situation-dependent and more research is needed to understand the importance of glutamine degradation in this process. GLS1 and GLS2 differ in their structure, motility, and regulatory mechanisms. GLS2 is associated with increased glutathione levels, and GLS2, like GLS1, can regulate the oxidative stress resistance properties of cancer cells [126]. Indeed, some studies have reported that tumor tissues from radioresistant patients exhibit significantly higher GLS2 levels than those from radiosensitive patients and that apoptosis, in response to radiation, is increased in GLS2-knockdown cancer cells [127]. Taken together, the significant involvement of GLS1 and GLS2 in the regulation of glutamine metabolism suggests the need for further study to explore the context-dependent divergent effects of GLS2 on tumorigenesis and its regulatory mechanisms in response to external stimuli.

Several studies have demonstrated that dysregulated GLS expression occurs due to the activation of oncogenes in tumor cells. c-Myc, a representative oncogene, has been shown to increase GLS expression and increased GLS2 expression has been shown to increase the expression of SNAIL, a transcriptional activator of EMT both *in vivo* and *in vitro* [128]. In addition, recent studies have identified the molecular alterations that modulate c-Myc. Growth-promoting signaling molecules, including mTOR complex 1 (mTORC1), drive the metabolic reprogramming of cancer cells that is required to support their biosynthetic needs for rapid growth and proliferation. Activation of the mTORC1 pathway has previously been shown to promote the anaplerotic entry of glutamine into the TCA cycle, via glutamate dehydrogenase (GDH) [129]. Moreover, mTORC1 activation stimulates the uptake of glutamine, although the mechanism is unknown. mTORC1 controls GLS levels through the ribosomal protein S6 kinase beta-1 (S6K1)-dependent regulation of MYC oncogene. S6K1 enhances the efficiency of MYC translation by modulating the phosphorylation of eukaryotic translation initiation factor 4B (eIF4B), which is critical for unwinding its structured 5′ untranslated region [130]. Recent research has shown that glutamine deprivation results in a decrease in STAT3 phosphorylation at serine 727, which enhances mitochondrial respiration and STAT3 tyrosine phosphorylation in malignant ovarian cancer cells. The inhibition of constitutive STAT3 phosphorylation represses the expression of its target genes, which include genes that regulate metastasis. Treatment with α-ketoglutarate results in STAT3 tyrosine phosphorylation, which restores the invasiveness of tumor cells. The involvement of STAT3 in glutamine metabolism has been exploited to inhibit glutaminolysis, following a study showing that knockdown GLS or GDH expression blocked STAT3-mediated EMT, migration, and invasion *in vivo* [131]. Recent research has shown that GDH is upregulated in colorectal cancer and in metastatic lesions. Moreover, knockdown of GDH significantly attenuated STAT3 phosphorylation and decreased vimentin and ZEB1 expression, while upregulating E-cadherin expression. These effects were reversed by exogenous GDH expression [132,133]. As a preceding step of glutaminolysis, glutamine uptake is mediated by the glutamine transporter, SLC1A5, which exchanges glutamine for another essential amino acid and has been reported to activate mTORC1. High glutamine utilization has been shown to contribute to cancer cell migration, partly by activating mTORC1 [134]. As glutamine utilization also increases lipogenesis by providing both acetyl-CoA and NADPH, the direct contribution of glutamine to de novo lipogenesis is particularly apparent under conditions of hypoxia or mitochondrial dysfunction, in which cells depend almost exclusively on the reductive metabolism of α-ketoglutarate to synthesize acetyl-CoA [135]. Glutamine-mediated lipogenesis and EMT induction, in turn, regulate the activation of AKT, which has been widely studied for its involvement in the migratory and invasive behavior of many cancer cell lines. These results indicate that the molecular signaling pathways involved in tumor cell malignancy increase the expression of genes related to glutaminolysis metabolism and lead to the acquisition of metastatic ability.

As discussed above, most studies of amino acid metabolism have focused on glutamine metabolism. Glutamine plays key roles in metabolic reprogramming, and some studies have indicated that glutamine metabolism may regulate tumor metastasis. Recently, however, research on other amino acid metabolites has also been reported. For example, upregulation of the solute carrier, solute carrier family 38 member 3 (SLC38A3), has been shown in metastatic tumor cells. Upregulated levels of SLC38A3 increased glutamine/histidine export. This induced the activation of PI3K/AKT/GSK3β/β-catenin signaling, which in turn, induced EMT and metastasis of non-small cell lung cancer (NSCLC) cells [136]. Once tumor cells detach from their primary site, their H^+^ gradient is reversed, leading to glutamine efflux and phosphoinositide-dependent kinase-1 (PDK1) activation. Recent research has shown that proline metabolism is also related to the EMT process and metastasis. The *in vivo* role of proline dehydrogenase (PRODH) activity in cancers has not yet been studied. However, PRODH expression is inversely correlated with spheroid size. Interestingly, it has been reported that c-Myc is a negative regulator of PRODH expression. C-Myc, which negatively regulates PRODH expression, clusters with metastasis size, rather than with the metastatic site, suggesting that PRODH inhibition may be independent of the organ in which the metastases form. These data suggest that targeting PRODH activity may be an effective strategy to control EMT processes and metastasis in breast cancer [137].

## 5. Clinical Significance of Metabolic Regulation in Cancer Therapy

Cancer therapies targeting metabolic pathways have been investigated for many years. Although some drugs have been successful, most have not, because of lack of efficacy or severe toxicity. In this section, we summarize the representative metabolic enzyme-targeting drugs that are either FDA-approved or are currently in trials and discuss their efficacy and toxicity (Table 1).

Benserazide, a specific inhibitor of hexokinase 2, has historically been used to treat Parkinson’s disease patients. However, it has recently been shown to inhibit the progression and metastasis of colon cancer cells by inhibiting MMP expression [138]. Although no clinical trials of benserazide as an anti-cancer treatment have been reported, its safety has been demonstrated in Parkinson’s disease patients, and therefore, it has the potential for use as an anti-cancer agent. Lonidamine, another specific inhibitor of hexokinase 2, has been tested in patients with prostate and breast cancer, but it did not show any significant clinical response at doses with low toxicity [139,140]. However, recent studies have suggested that the combination treatment of cisplatin and lonidamine is effective in advanced ovarian cancer patients, with 80% of patients showing a complete or partial response [141]. Although there are four isoforms of HKs from HK1 to HK4, each HK has unique functions and regulatory mechanisms which need different molecules for specific inhibition [142]. These studies suggest that targeting HK2 is a promising anti-tumor strategy and the further development of combination treatments may increase its therapeutic efficacy. Gossypol, also known as AT-101, is an LDHA inhibitor that has shown significant anti-tumor effects in various types of tumor cells [143,144]. It induces genotoxicity-mediated apoptosis and suppresses the invasion of cancer cells [145,146]. However, some phase II clinical studies have shown that the use of gossypol to overcome chemoresistance did not meet the therapeutic efficacy requirements and further studies are needed before its clinical use. Mutations in isocitrate dehydrogenase (IDH) have been identified in many types of cancers, including glioblastoma and acute myeloid leukemia [147,148]. Mutant IDH in cancer cells catalyzes the reduction of α-ketoglutarate (α-KG) to D-2-hydroxyglutarate (D-2HG), whereas wild-type IDH catalyzes the decarboxylation of isocitrate to α-KG. Since mutant IDH functions as an oncogenic enzyme, treatments that target the enzyme have been tested in cancer patients. Most drugs targeting the enzyme are currently in clinical trials. They are expected to reduce cancer cell proliferation and metastasis, because increased D-2HG has been shown to induce EMT through mechanisms, such as DNA hypermethylation, TET methylcytosine dioxygenase 2 (TET2) dysfunction, and increased HIF-1α and serum Vesicular endothelial growth factor (VEGF) levels [149,150,151]. As discussed above, a significant amount of the research on metabolic regulation, to determine the clinical significance of anti-cancer therapies that target these processes, has focused on the regulation of glycolytic enzymes. Further research on the combination of classic therapies and a deeper understanding of glycolytic enzyme regulation will facilitate the development of therapies with enhanced efficacy.

There have been numerous studies investigating lipid oxidation-induced cell stress and its inhibitors for the control of tumor progression. COX-2 inhibitors are used as anti-inflammatory drugs to treat syndromes induced by excessive inflammatory responses, including cardiovascular, regenerative, and central nerve diseases [152]. Although many kinds of COX-2 inhibitors have been tested, only a few have shown clinical significance. Etodola has been shown to be effective when administered to uterine, breast, and gastric cancer patients [153,154,155]. It prevents metastatic progression, which provides additional support for the role of COX-2 in EMT induction. Conversely, some clinical trials have shown that the COX-2 inhibitor, celecoxib, is effective at controlling tumor progression [156,157]. This discrepancy may be due to the differences between these two inhibitors and their underlying molecular mechanisms. However, it cannot be denied that COX-2 inhibition helps control cancer progression. This has been demonstrated in individual studies and in a meta-analysis, which showed that colorectal cancer patients using COX-2 inhibitors, such as celecoxib, rofecoxib, and etoricoxib, showed improved survival after chemotherapy [158,159]. Finally, PPAR agonists and antagonists have been widely used to control metabolic disorders, including diabetes, non-alcoholic fatty liver disease, and obesity [160]. Recently, clinical trials have demonstrated the potential of using PPAR agonists as anti-tumor agents. Efatutazone, an oral PPARγ agonist, shows biological activity, with acceptable toxicity, when used in combination with classic anti-tumor therapies [161,162]. Another PPARγ agonist, pioglitazone, has been reported to induce a significant increase in molecular response in leukemia patients, in combination with imatinib [163]. In addition, pioglitazone treatment suppresses thyroid cancer progression and metastasis, with acceptable levels of toxicity [164]. Despite its significant roles in tumor growth and metastasis, FASN inhibitors have not yet been investigated in clinical trials. Together, these studies indicate that EMT-regulating lipid metabolism enzymes are promising clinical targets for controlling cancer progression and that further clinical trials assessing the efficacy of such drugs are warranted.

Glutaminolysis is an important step in the TCA cycle to supply α-ketoglutarate. To block the TCA cycle in cancer cells, bis-2-(5-phenylacetamido-1,3,4-thiadiazol-2-yl)ethyl sulfide (BPTES) and CB-839 have been developed as inhibitors of glutaminase [165]. Treatment with both siGLS1 and BPTES decreases ATP levels, glucose uptake, and the number of invading cells [166]. Furthermore, BPTES and CB-839 significantly reduce basal and maximal oxygen consumption rate, which indicates a decrease in mitochondrial oxidative phosphorylation [167]. In addition, BPTES was referred to inhibit only GLS1 and CB-839 was referred to inhibit both GLS 1 and 2 [165,168] These results suggest a possible application of glutaminolysis inhibitors for EMT suppression in cancer cells with underlying molecular events by the treatment. Epigallacatechin-3-gallate (EGCG) is a natural product that inhibits GDH. It has been shown to improve the efficacy of radiotherapy in breast cancer patients, with acceptable levels of toxicity [169]. As EGCG is found in green tea extracts, it is expected to be safe and feasible to investigate in clinical trials [170]. In addition, a clinical study has shown that EGCG regulates FASN expression and has effects on tumor control [171]. Although there are few reports on clinical trials of molecules that regulate amino acid metabolic enzymes, further studies on the regulation of amino acid metabolism in cancer cells are warranted.

## 6. Conclusions

**Figure 1:**
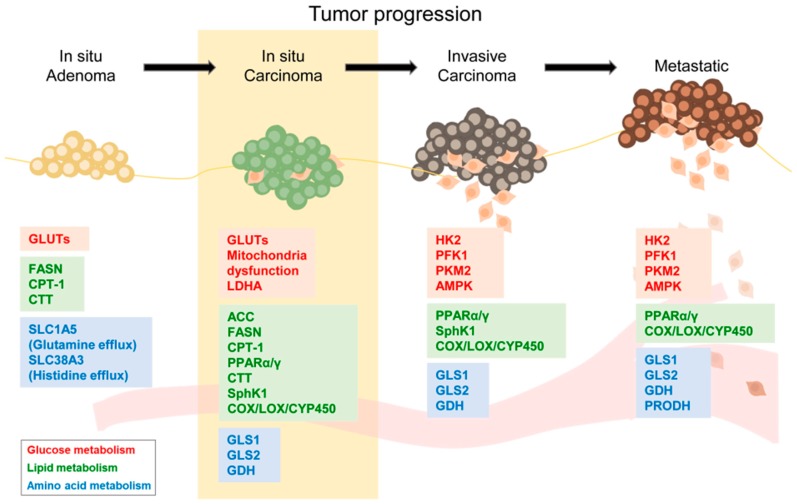
Metabolic factors in epithelial–mesenchymal transition (EMT) and metastasis. A diagram summarized the effects of metabolic factors in EMT and metastatic progression of tumor cells. The color of boxes indicated the related metabolic processes. The yellow box indicates the EMT process during cancer progression.

In this review, we summarized the common metabolic reprogramming processes in cancer cells, including activated glycolysis, lipogenic states, and dysregulated amino acid utilization. Metabolic reprogramming in cancer cells induces the utilization of cellular energy sources and regulates the expression of EMT-related genes. A graphical summary of previous studies on the roles metabolic reprogramming in EMT and metastasis is portrayed in Figure 1. It provides information that may be useful for the application of metabolic regulation to suppress tumor progression.

In addition, we discussed the clinical trials investigating the efficacy of metabolic regulators as anti-cancer therapies that have shown significant effects and acceptable levels of toxicity. Most of these trials involved the administration of the new drug in combination with a classic anti-cancer drug since sole treatments show minimal anti-cancer effects. This suggests that metabolic regulation has latent toxicity and a delicate approach is needed to utilize a metabolic regulator only at doses with acceptable levels of toxicity. In this context, some of these metabolic regulators have been used to treat patients with other diseases, including diabetes, obesity, and menopause, which removes any concerns about clinical toxicity. Taken together, further clinical research on the application of metabolic regulators as anti-cancer adjuvants are necessary for the development of therapies with enhanced efficacy.

## Figures and Tables

**Table 1:** List of metabolic regulators with clinical significance for anti-cancer therapy.

Related Metabolism	Drug	Function	Reference
Glucose	Benserazide	Hexokinase2 inhibitor	[138]
	Lonidamine		[139,140,141]
	Gossypol (AT-101)	LDHA inhibitor	[143,144,145,146]
Lipid	Etodola	COX-2 inhibitor	[153,154,155]
	Celecoxib		[156,157]
	Etoricoxib		[158,159]
	Efatutazone	PPARγ agonist	[161,162]
	Pioglitazone		[163,164]
Amino acid	BPTES	GLS inhibitor	[165,166,167]
	CB-839		[165,167]
	Epigallacatechin-3-gallate (EGCG)	GDH inhibitor	[169,170,171]
